# Electrical bioimpedance spectral markers distinguish balance ability in older adults without provoking fall risk

**DOI:** 10.1186/s12984-026-02155-8

**Published:** 2026-09-11

**Authors:** Abdelakram Hafid, Mia Folke

**Affiliations:** Department of Computer Science and Engineering, Mälardalen University, Västerås, Sweden

**Keywords:** Electrical bioimpedance, Postural control, Spectral analysis, Muscle impedance, Tandem standing, Fall risk assessment

## Abstract

**Background:**

Balance assessment in older adults is central to fall-risk evaluation and rehabilitation planning, yet widely used clinical tests such as the single-leg stance require postures most likely to provoke a fall. A method capturing neuromuscular contributions to postural control during safer, static postures would meaningfully advance clinical and home-based assessment.

**Method:**

We investigated whether electrical bioimpedance (EBI) recorded over the extensor digitorum longus (EDL), a muscle contributing to fine ankle-level postural adjustments, carries spectral signatures reflecting balance ability without provocative testing. Twenty-eight community-dwelling adults aged 60 to 74 years were stratified into two balance-ability groups by single-leg-stance performance, and EBI was recorded using a tetrapolar configuration during sitting, stable standing, and tandem standing. After standardized preprocessing, signals were analyzed in the time and frequency domains using spectral and time-frequency decomposition with peak-band and transient-event detection. From 61 candidate features, a non-redundant primary set of 25 was retained through predefined reduction rules and correlation screening. Between-group differences were evaluated using activity-adjusted, feature-wise generalized estimating equations, validated by participant-level permutation testing, with multiple-comparison control across the retained feature set.

**Results:**

Physiologically meaningful spectral power was concentrated within 0–10 Hz across all three postures. After activity adjustment, two frequency-domain features remained significant under false discovery rate correction and one survived Bonferroni correction. Participants with better balance exhibited stronger oscillatory organization of the EBI signal, reflected in greater power concentration within identifiable spectral peaks and a greater peak count, whereas participants with poorer balance showed a less peak-dominated, more broadband profile with larger and more irregular low-frequency amplitude fluctuations.

**Conclusion:**

Low-frequency spectral features of muscle EBI, recorded during safe static postures, distinguish balance ability in older adults and reveal differences in the temporal organization of distal postural-control activity. These findings support EBI-derived spectral markers as candidate muscle-level biomarkers of balance-control quality, offering a path toward safer, sensor-based assessment that does not require older adults to adopt postures associated with increased fall risk. The approach is well suited to integration with rehabilitation monitoring and could complement existing clinical balance evaluations in both supervised and unsupervised settings.

## Introduction

Falls are a major global health problem. The World Health Organization estimates that more than 684,000 people die from a fall each year, and that 30% of people over 65 years of age and 50% of people over 85 years of age fall at least once a year [1–3] For people of almost all ages, falls are the leading cause of injury [4, 5]. Muscle strength and balance control, the capacities most closely related to fall risk, decline with age [6].

The World guidelines for falls prevention and management for older adults (2022) are the current international reference for assessing this risk, recommending gait speed for stratification and, within a multifactorial assessment, balance tasks such as tandem and one-leg stance together with the chair stand test or handgrip strength [2]. Such measures capture functional performance and gross strength rather than the neuromuscular activity generating postural corrections; questionnaire-based tools have documented limitations [7], and no single physical test predicts fall risk reliably [8]. Moreover, several tests in common use, including the 30-second Sit-to-Stand test [9], the Four-Square Step Test [10], and the Functional Reach test [11], themselves introduce a risk of falling, because they require individuals to perform at or near their maximum capacity.

The same guidelines were the first to address sensor technologies explicitly, conditionally recommending telehealth and smart-home systems combined with exercise while concluding that current evidence does not support wearables for fall prevention [2]. That conclusion concerns wearables used within prevention programmes; no sensor-based method appears among the recommended assessment tools, and e-technology was identified as an area where evidence remained too limited for strong recommendations [2]. Whether wearable sensing can contribute to fall risk assessment therefore remains open. A systematic review of wearable sensor-based fall risk assessment in older adults found that the studies meeting its inclusion criteria used only pressure sensors, accelerometers, gyroscopes, and inertial three-dimensional motion sensors, all of which capture the mechanics of body movement rather than muscle activity [12].

Inertial measurement devices, however, have several limitations for detecting early age-related decline. Because they capture segmental kinematics (acceleration, angular velocity, and orientation) rather than neuromuscular activity, they cannot characterize the status or activation of the muscles producing a movement [13]. They therefore describe the mechanical output of a task rather than the underlying muscle-control strategy and may fail to detect early or compensated declines in which movement output is preserved while neuromuscular control is already altered; their accuracy is further affected by sensor placement and attachment, including movement of the sensor with the overlying soft tissue, and by drift or integration error when signals are used to estimate velocity or position [14, 15] .

Neuromuscular activity can instead be assessed directly using electromyography (EMG), a well-established and extensively validated method for characterizing age-related changes in muscle function. Surface and intramuscular EMG have been used to document the motor-unit remodeling that accompanies ageing, including motor-unit loss, enlarged and more complex motor-unit action potentials arising from collateral reinnervation, reduced motor-unit discharge rates at matched force levels, and increased antagonist co-activation during movement [16, 17]. The validity of these indices is supported by convergent evidence from independent approaches, including motor-unit number estimation, which shows progressive reductions in motor-unit number in older adults, and studies combining electrophysiological estimates with imaging-derived measures of muscle cross-sectional area [18, 19]. EMG-derived amplitude measures show good relative test-retest reliability for standardized isometric tasks in older adults, although their absolute reproducibility is considerably poorer, and reliability depends on consistent electrode placement and signal normalization [20, 21]. Surface EMG is nevertheless constrained by well-documented factors: activity from neighbouring muscles contributes to the recorded signal, which is most problematic where several small muscles lie close together, and signal amplitude depends on subcutaneous fat and skin thickness and on electrode size, spacing and position relative to the innervation zone, so amplitudes are not comparable between individuals or sessions without normalization [22, 23]. These constraints are most severe for small distal muscles during low-level, intermittent activation, as in quiet standing.

The extensor digitorum longus (EDL) illustrates this difficulty. It is a small muscle of the anterior compartment, lying immediately adjacent to tibialis anterior and the fibularis muscles, and its activation during static postures is minimal and intermittent. Electrical bioimpedance (EBI) offers a complementary approach: rather than recording the electrical drive to the muscle, it measures the electrical properties of the tissue, which vary with the changes in geometry and composition accompanying contraction and fluid redistribution, enabling monitoring of muscle activation and analysis of physical activity [24–26], muscle composition [27], and hydration [28]. It uses the same class of surface electrodes, is compatible with low-power wearable instrumentation, and does not depend on innervation-zone geometry. Its interpretation is nevertheless affected by inter-individual differences in subcutaneous fat and skin thickness, tissue water content, electrode positioning, and electrode-skin interface impedance [25, 29]. EBI is therefore examined here as a complement to established methods rather than as a replacement for them.

We have shown in previous studies that dynamic lower-limb exercises such as squats and lunges produce distinct and repeatable EBI signal characteristics [30], and that these characteristics differ across age groups [31]. In the first of these studies, the recorded signals showed reproducible waveforms with identifiable movement phases, from which time-domain features describing contraction amplitude (position amplitude and baseline-to-peak amplitude) and neuromuscular coordination (the number of signal fluctuations) were extracted; the fluctuation feature, in particular, reflected the balance and coordination demands of the movement [30]. In the second study, the EDL muscle showed a consistent, monotonic age-related decline across its impedance features, including an approximately 17% reduction in resting impedance magnitude during relaxed standing from the young to the old group, in line with the age-related loss of muscle mass, whereas the quadriceps muscle followed a more variable, non-monotonic pattern [31]. Together, these findings indicate that muscle EBI is sensitive both to the mechanical demands of a task and to age-related physiological change, and that the EDL muscle carries a particularly consistent age-related signal.

Together, these considerations reveal a gap in current fall-risk assessment: conventional balance tests require provocative postures, whereas inertial sensors avoid this risk but cannot access the underlying neuromuscular activity. Electrical bioimpedance can capture muscle-level activity non-invasively, yet previous EBI studies, including our own, have used only time-domain, magnitude-based features and have not examined whether EBI can distinguish balance ability, or whether the frequency-domain organization of the signal is informative, during safe static postures. Closing this gap would enable safer, sensor-based assessment suitable for home-based and unsupervised settings. Accordingly, the aim of this study was to characterize EBI signals of the EDL muscle during static postures (sitting, stable standing, and tandem standing) and to assess whether EBI features differ by balance ability, as defined by single-leg-stance performance.

## Materials and methods

The study was approved by the Swedish Ethical board Dnr 2025-00469-01 and adhered to the Declaration of Helsinki. All participants received oral and written detailed information regarding the study, and each participant provided written informed consent before the sensor measurements started.

### Participants

Participants were recruited from a fall-prevention training programme arranged by a local sports club. All individuals attending the programme were invited to take part, and the following criteria were applied. Participants were included if they were aged 60 years or over, were attending the fall-prevention training programme, and were able to stand unsupported and attempt single-leg stance without manual assistance. Participants were excluded if they were unable to follow the verbal instructions required for the measurement protocol or were unable to fully complete the training programme due to any physical, cognitive, or other impediment. No formal medical screening was undertaken, and no restrictions were placed on comorbidities, medication use, or prior fall history. Twenty-eight older adults (60–74 years, mean age 65.6 years; 10 women, 18 men) were recruited on this basis. Single-leg stance performance was subsequently used to stratify participants into two functional groups.

EBI measurements were performed on the leg with the poorer single-leg stance performance (hereafter, the “sensor leg”), identified by testing both legs and selecting the leg on which the shorter stance duration was achieved. This leg was selected for two reasons. First, postural control is least well maintained on this limb, so balance limitations are most readily expressed; recording from it therefore increases the sensitivity of the 60 s criterion and reduces the ceiling effect that would arise if participants were tested on their better leg. Second, defining the sensor leg by measured performance standardizes the procedure across participants and avoids reliance on self-reported limb preference.

Participants who maintained single-leg stance on the sensor leg for 60 s were assigned to Group Passed (P). Participants who were unable to maintain single-leg stance for 40 s were assigned to Group Failed (F). Participants with intermediate performance (≥ 40 s but < 60 s) were excluded a priori to avoid an intermediate class; three participants met this criterion in the present cohort and were excluded, leaving 25 participants for analysis (*P* = 14, F = 11). Stance duration was recorded in 10 s intervals. Within Group F, six participants maintained stance for less than 10 s, two for 10 to 20 s, and three for 20 to 30 s. All 14 participants in Group P reached the 60 s criterion, at which point the test was stopped. No participant stood between 30 and 60 s, so the two groups were clearly separated, and a binary classification was used rather than a continuous analysis of stance duration.

### Instrumentation and protocol

**Fig. 1:**
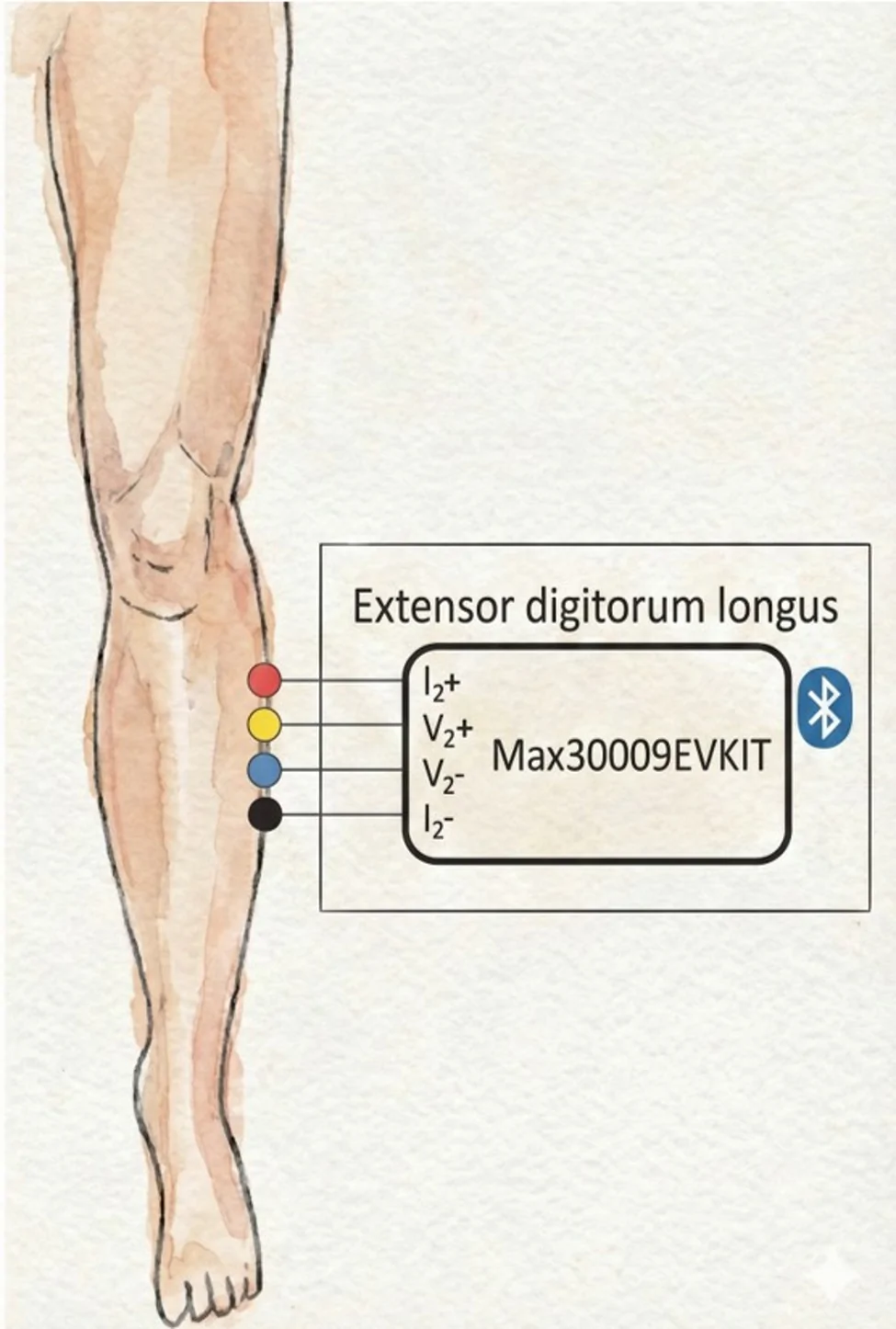
Schematic representation of the tetrapolar EBI sensor configuration. Four Ag/AgCl gel electrodes are positioned along the EDL muscle of the sensor leg

The Max30009EVKIT EBI device (Analog Devices, Inc., Wilmington, MA, USA) was used for data collection. The devices were carried in a bag, fastened around the participants’ waists. The sensors were placed on the participants’ *sensor leg*. A tetrapolar configuration of the sensors was used: four Ag/AgCl gel electrodes positioned on the EDL muscle to facilitate the EBI measurements, as shown in Fig. 1.

The injected alternating current had a frequency of 50 kHz, with an amplitude of 1.28 mA. The maximum sampling rate was set to 390 Hz. The measurement setup is presented in detail in our previous study [30].

**Fig. 2:**
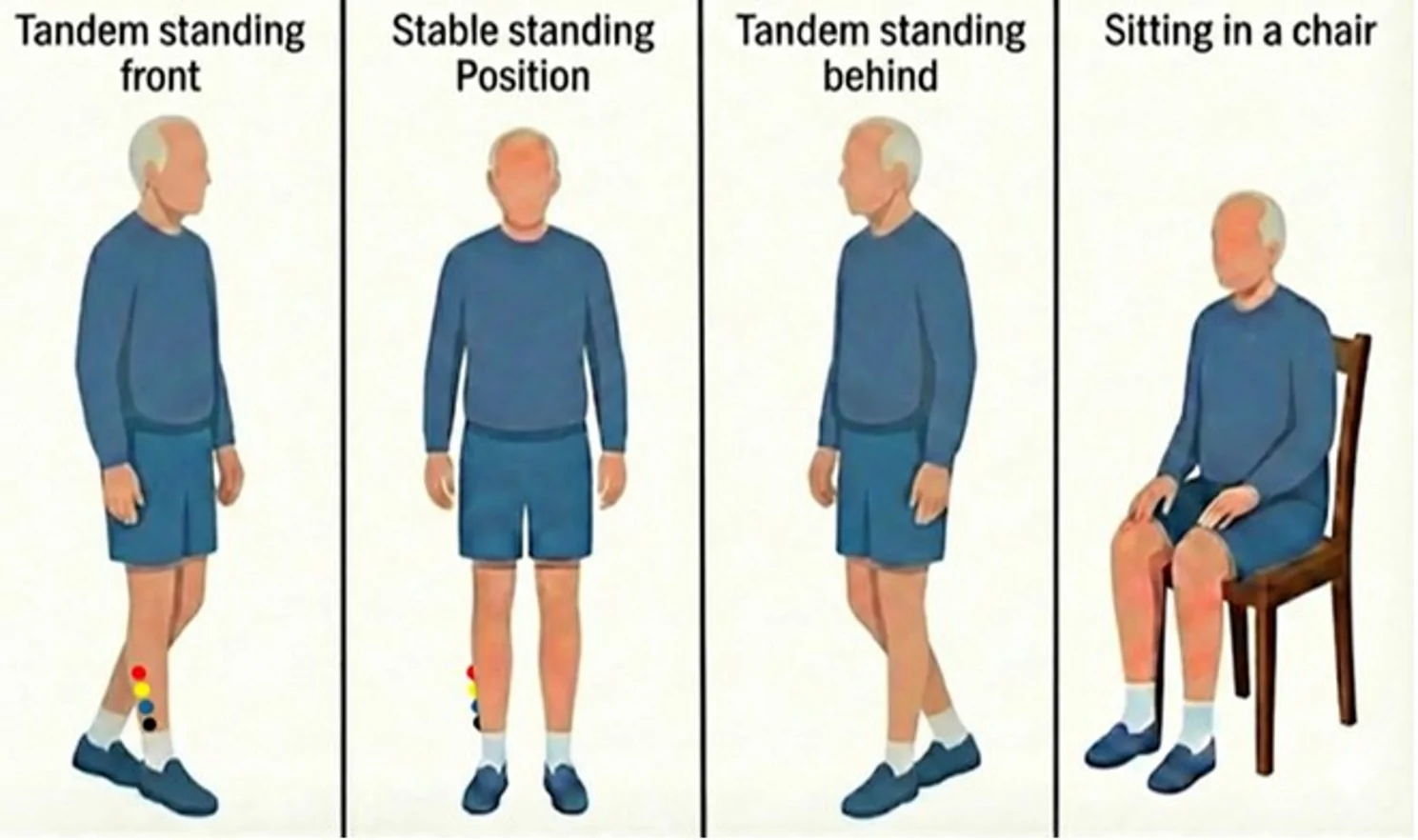
Participant activity protocol: *TS_F*,* St_P*,* TS_B and Sit_P*

EBI data were collected while the participants assumed different positions across four segments. For the first segment, the participant was asked to stand with the sensor leg in front of the other foot, heel to toe (*Tandem Standing Front* (TS_F)) for 40 s. After that the participant was asked to stand stable with the feet a little apart (*Stable Standing Position* (St_P)) for 40 s. Then, the participant was asked to perform tandem standing with the sensor leg behind for 40 s (*Tandem Standing Behind* (TS_B)), and finally the participant was asked to sit in a chair (*Sitting Position* (Sit_P)) for 40 s (Fig. 2). Each segment was recorded once per participant, in the fixed order described above, and no segment was repeated within or across sessions. Feature extraction was therefore based on a single continuous recording per segment rather than on an average across repeated trials.

Participants received verbal and visual instructions on how to execute and when to start each segment. No practice or familiarization attempts were performed; each segment was recorded on the first attempt, and all participants executed the segments correctly, so no segment required repetition. Rest periods between segments were not fixed in duration: participants proceeded to the next segment when they indicated they were ready, which in all cases was within 1 min. Both researchers monitored safety and documented notable balance-correction behaviors for qualitative comparison; these observations were not used in feature computation or statistical modeling.

### Data processing

The collected data were analyzed and processed offline using a custom program implemented in MATLAB 2024a (The MathWorks, Inc., Natick, MA, USA) and Python (version 3.12.1) with established scientific computing libraries (pandas, NumPy, SciPy). For each activity trial, the central 30 s of the recorded EBI time series were retained to reduce start/stop transients and to ensure comparable steady-state windows across participants. Amplitude min-max scaling was not applied because it distorts signal power and biases spectral features; instead, amplitude comparability across participants was achieved using power ratios and fractional metrics that are invariant to absolute amplitude scaling.

*Spectral analysis and time-frequency decomposition.* To characterize the spectral content of the EBI signals, a short-time Fourier transform (STFT) was applied using a Hann window with fixed parameters across all participants to ensure feature comparability. The window duration was 5.0 s, providing a nominal spectral resolution of approximately $\:1/T\:\approx\:\:0.2\:Hz$ while maintaining temporal sensitivity to transient postural adjustments. Window overlap was 80%, yielding a time step of approximately 1.0 s between successive spectra. The FFT length was fixed at *N*_*FFT*_ = 8192 (zero-padding as needed), resulting in a frequency-bin spacing of $\:\varDelta\:f\:\:=\:\:{f}_{s}/{N}_{FFT}\:\approx\:\:0.048$ Hz for *fs* = 390 Hz [32].

An analysis bandwidth of 0–10 Hz was chosen. The choice was motivated by the physiological frequency content of postural control [33]. Postural sway during quiet standing exhibits dominant frequencies between 0.1 and 2 Hz [34], while corrective muscle activations associated with balance maintenance typically occur below 10 Hz [35, 36]. Visual inspection of power spectral density estimates across all participants confirmed that signal energy was concentrated between 0 and 10 Hz, with negligible power above this range. The resulting complex spectrogram S(*f*,* t*) was transformed into a power spectrogram using $\:P(f,t)\:\:=\:\:\left|S\right(f,t\left)\right|^{2}$, and subsequent analyses focused exclusively on the 0–10 Hz range.

*Participant-adaptive spectral band definition.* To avoid imposing arbitrary frequency cutoffs that may not align with individual participants’ physiological characteristics, we implemented a participant-adaptive band splitting approach. For each trial, a time-averaged power spectrum was computed by averaging P(*f*,* t*) across the temporal dimension. The spectral median frequency ($\:{f}_{med}$) was then calculated as the frequency at which cumulative power reached 50% of the total integrated power in the 0–10 Hz range [34, 37]. This $\:{f}_{med}$ served as an individualized split point, dividing the spectrum into low-band [0, $\:{f}_{med}$] and high-band [ $\:{f}_{med}$, 10] regions that track where each participant’s spectral energy is concentrated. This adaptive approach accounts for inter-individual variability in muscle activation patterns and postural control strategies without requiring a priori assumptions about optimal frequency boundaries.

*Spectral feature extraction.* From the time-averaged spectrum within 0–10 Hz, we computed the following features: (i) spectral centroid (power-weighted mean frequency, representing the center of mass of the spectrum), (ii) low-and high-band powers, defined as frequency-integrated spectral power within each band and expressed on a decibel scale as $\:{P}_{\mathrm{d}\mathrm{B}}\:=\:10{\mathrm{l}\mathrm{o}\mathrm{g}}_{10}\left(P\right)$ (arbitrary units); in parallel, fractional band powers were computed as amplitude-invariant descriptors, (iii) fractional power in each band ($\:\:frac\_low=\:{P}_{low}\:/\:({P}_{low}\:+\:{P}_{high})$ and $\:frac\_high=1\:-\:frac\_low$, providing amplitude-independent metrics) [38, 39]. Finally, (iv) spectral entropy, calculated as the Shannon entropy of the normalized power spectrum ($\:{H}_{spec}=-\sum\:({p}_{i}\mathrm{l}\mathrm{o}\mathrm{g}_{2}({p}_{i}))$, where $\:{p}_{i}=\:\:P\left({f}_{i}\right)\:/\:\sum\:P\left({f}_{i}\right)\:$) [40]. Spectral entropy quantifies the concentration of power across frequencies, with smaller values indicating energy concentrated in fewer frequency bins and greater values indicating a more diffuse broadband spectrum [40].

*Transient event detection from deviation maps.* To identify and quantify transient episodes of atypical spectrotemporal activity that may correspond to balance corrections or instability events, we applied a deviation-based event detection algorithm. The power spectrogram was converted to decibel scale ($\:10\:{log}_{10}\left(P\right(f,t\left)\right)$). For each frequency bin, a baseline was defined as the temporal median and subtracted to yield a median-subtracted deviation map D(*f*,* t*). The deviation map was collapsed across frequency by computing the frequency-integrated absolute deviation at each time-point: $\:E\left(t\right)\:\:=\:\:\varDelta\:f{\sum\:}_{f}\:\left|D\right(f,t\left)\right|$, where *Δf* is the frequency bin width. To establish a robust threshold for event detection, E(*t*) was converted to a robust z-score using the median and median absolute deviation (MAD): $\:z\left(t\right)\:=\:0.6745\left(E\right(t)\:-\:\mathrm{m}\mathrm{e}\mathrm{d}\mathrm{i}\mathrm{a}\mathrm{n}(E\left)\right)\:/\:\mathrm{M}\mathrm{A}\mathrm{D}\left(E\right)$, where the factor 0.6745 converts MAD to an estimate of the standard deviation under Gaussian assumptions [41]. Time intervals where z(*t*) exceeded 3.0 [41] were labeled as events, and temporally contiguous above-threshold frames were merged into discrete event intervals. For each trial, three event descriptors were computed: (i) *event_count*, the number of discrete event intervals; (ii) *event_time_coverage*, the fraction of the analyzed 30 s epoch occupied by events; and (iii) *event_max_z*, the maximum robust z-score observed during the epoch, indicating the magnitude of the most prominent deviation.

*Peak-band feature extraction.* To characterize prominent spectral peaks and their structure, the time-averaged power spectrum (0–10 Hz) was analyzed using a local maximum detection algorithm. Peaks were identified using *findpeaks* function in the MATLAB Signal Processing Toolbox, with a minimum prominence threshold set to 5% of the maximum spectral value and a minimum separation of 0.5 Hz, which reduces the detection of noise**-**driven or artifactual maxima rather than true physiological peaks [42]. Up to three most prominent peaks were retained for each trial. When fewer than three peaks met detection criteria, the corresponding peak features (peak2_*, peak3_*) were undefined, resulting in physiologically meaningful missing data. For each detected peak, descriptors were computed including the center frequency (*f*_*0*_) and the bandwidth ($\:bw\:\:=\:\:{f}_{R}\:-\:{f}_{L}$), defined at the full-width at half-maximum (FWHM) where power falls to 50% of peak power [43]. Additional metrics included peak power (dB) and the fraction of total 0–10 Hz power contained within the peak bandwidth. Summary metrics included the number of detected peaks (*n_peaks*) and the total fraction of power captured by all detected peaks (*peaks_total_frac_power*). The complementary background spectrum, comprising all frequency bins not assigned to any peak, was characterized by its integrated power (*bg_power_dB*) and fractional power contribution ($\:bg\_frac\_power\:\:=\:\:1\:-\:peaks\_total\_frac\_power$). To quantify the complexity and diffuse nature of this underlying spectral component, spectral entropy (*bg_spec_entropy*), was calculated, providing a robust measure of the signal’s irregularity beneath discrete oscillatory peaks [43].

*Time-domain feature extraction.* To complement frequency-domain characterization, we derived a parsimonious set of time-domain descriptors from standardized 30 s EBI epochs for each activity, leveraging fixed-length segmentation to obtain statistically reliable and compact representations of signal dynamics. The selection of a 30 s window reflects a trade-off between temporal resolution and estimation robustness, as excessively short windows lead to unstable feature estimates, whereas longer windows may obscure transient physiological variations [44]. Moreover, the use of 30 s epochs is consistent with established practices in physiological signal analysis, where such durations provide sufficient data to ensure stable feature extraction while maintaining interpretability across time-varying biological processes [45]. To preserve physiologically interpretable absolute impedance information while avoiding redundancy with detrending, features were computed on two complementary representations of the same epoch: (i) the raw segment to quantify baseline level, and (ii) a low-frequency (LF) dynamic component obtained by zero-phase low-pass filtering at 10 Hz (4th-order Butterworth; applied with forward–reverse filtering) [46] followed by removal of the DC and linear trend (first-order detrending). From the raw segment, we extracted baseline features reflecting absolute impedance level (mean, median, minimum, maximum). From the LF detrended signal, we extracted dynamic descriptors emphasizing variability and waveform structure independent of baseline drift, including dispersion and amplitude range (standard deviation, variance, root-mean-square, interquartile range, peak-to-peak amplitude), distributional shape (skewness, kurtosis), and activity/complexity measures. Activity measures included the zero-crossing rate computed on the detrended LF signal as the proportion of sign changes between consecutive samples [44, 47], total LF energy (sum of squared samples), and roughness metrics derived from the first difference: mean absolute derivative, standard deviation of the first derivative, and line length per second, defined as $\:{\sum\:}_{n}\mid\:x(n+1)-x\left(n\right)\mid\:/T$ [48], where * T* is epoch duration (s). This combined baseline dynamic feature strategy yields a time-domain characterization complementing the spectral metrics with interpretable measures of amplitude dynamics and temporal structure [47].

### Feature analysis and redundancy screening

*Initial feature pool and extraction framework.* Signal processing and feature extraction yielded an initial pool of 61 candidate features distributed across three complementary feature families: (i) 15 global spectral and transient event descriptors derived from STFT based time-frequency analysis (quantifying spectral distribution, spectral concentration, and transient event statistics), (ii) 30 peak-band and background features derived from spectral decomposition (characterizing oscillatory structure, dominant frequencies, and aperiodic components), and (iii) 16 time-domain descriptors computed from standardized 30 s epochs using a complementary baseline dynamic strategy: four baseline level features from the raw segment and 12 LF dynamic features (capturing amplitude variability, distributional shape, derivative-based roughness, zero-crossing rate, and signal energy). This comprehensive feature set was designed to capture multiple physiological dimensions of neuromuscular control during balance‑related tasks, including baseline muscle impedance state, oscillatory activation patterns, spectral organization, transient corrective responses, and signal complexity.

*Feature reduction strategy.* To improve interpretability and reduce the multiple-comparison burden, we constructed a non-redundant primary feature set using predefined reduction rules based on deterministic mathematical relationships and physiological redundancy [49, 50]. The reduction protocol systematically eliminated: (i) non-feature metadata fields (participant identifier, participant‑Group labels, activity codes, and segment timing variables); (ii) exact algebraic complements where two features are deterministically related by a complement relationship (for example for fractional power, $\:frac\_high=1-frac\_low$; $\:bg\_frac\_power=1-peaks\_total\_frac\_power$), retaining only one representative from each complement pair; (iii) algebraically dependent peak-geometry fields (e.g., *f*_*L*_ and *f*_*R*_, and the quality factor $\:Q={f}_{0}/bw$) were removed; peak structure was represented by *f*_*0*_, *bw*, and fractional power; (iv) strictly derived time-domain variables where one feature is deterministically computed from another for a fixed 30 s epoch length ($\:variance=SD^2$; $\:peak2peak\:range=max-min$; $\:signal\:energy=N\cdot\:RMS^2$ with constant $\:N=\:(fs\cdot\:30\:s)+1=\mathrm{11,701}$ samples per epoch [*fs* = 390 Hz; inclusive endpoint indexing]) [49]; and (v) duplicate constructs appearing across files, prioritizing the most robust and physiologically interpretable representative when multiple features captured the same underlying construct.

*Rationale for feature retention.* Feature retention was guided by three principles. (i) Mathematical non‑redundancy was enforced by removing deterministic relationships that would introduce perfect multicollinearity [50, 51]. (ii) Physiological interpretability was prioritized by retaining features that map to distinct aspects of neuromuscular control during balance‑related tasks, specifically baseline impedance state, amplitude variability, spectral concentration, dominant oscillatory structure, transient event characteristics, and signal roughness [52, 53]. (iii) Complementary information dimensions were preserved by ensuring that the retained feature set spanned time‑domain, frequency‑domain, and time‑frequency representations without substantial overlap in the physiological processes being examined.

*Final primary feature set.* The resulting primary set comprised 25 features organized into three functional categories: six global spectral and event features (spectral centroid, low-band fractional power, spectral entropy, event count, event time coverage, and maximum event z-score), 12 peak-band and background features (number of peaks, total peak fractional power, total power in dB, background power in dB, background spectral entropy, and peak descriptors). Peak descriptors included for the dominant peak (*Peak 1*) the center frequency, bandwidth, and fractional power; and for *Peaks 2* and *3* (when present) the center frequency and fractional power. Finally, seven time-domain impedance descriptors were retained using a baseline dynamic strategy: raw mean impedance (*RAW_mean*) to represent absolute baseline level, and six LF dynamic descriptors (*LF_peak2peak*, *LF_iqr*, *LF_meanAbsDeriv*, *LF_zeroCrossRate*, *LF_skewness*, *LF_kurtosis*). This 25-feature set was designated as the primary analytical target for inferential statistical testing. The complete 61-feature pool was retained for exploratory sensitivity analyses but was not used for primary inference to limit inflation of family wise error rates.

*Correlation screening among retained features.* After deterministic redundancy reduction, we performed a post‑reduction verification to ensure the 25 primary features were not trivially redundant [50]. For this, we computed pairwise Spearman rank correlations $\:\left(\rho\:\right)$ [54, 55] on participant‑level, activity‑residualized values (within‑activity mean subtraction, followed by within‑participant averaging, yielding one activity‑adjusted value per participant per feature). We prespecified a flagging threshold of $\:\left|\rho\:\right|>0.80$ to identify pairs with potential redundancy [50, 54]. When a pair exceeded the threshold, both features were retained if they represented distinct physiological constructs (e.g., peak‑structure vs. amplitude/roughness domains) or different representational domains (time vs. frequency); flagged pairs were documented to aid transparent interpretation and future multivariate modeling. This screening serves as a verification layer; it does not alter the inference framework. Primary statistical analyses were separate univariate models per feature with multiplicity control across the 25 tests; therefore, inter‑feature correlations do not bias coefficient estimation in our hypothesis tests.

*Feature interpretation framework.* To facilitate interpretation and ensure transparent reporting, each of the 25 primary features was described along two dimensions: (i) the signal‑processing definition (mathematical computation), and (ii) statistical considerations (e.g., boundedness, discreteness, skewness/outlier sensitivity, and handling of missingness). These descriptors are reported in Table 1. The purpose was to document how each feature is computed and any distributional properties relevant to modeling and visualization. In discussion sections, these feature descriptors are used to structure the interpretation and to motivate physiologically grounded hypotheses.

**Table 1:** Primary (non‑redundant) 25‑feature set used for inferential testing

Feature	Signal-processing definition	Statistical considerations
*f_centroid_Hz*	Spectral centroid (power-weighted mean frequency) within 0–10 Hz.	Continuous; can be outlier-sensitive when spectra are flat or multi-modal.
*frac_low*	Fraction of total 0–10 Hz power in the individualized low band [0, *f*_*med*_].	Bounded [0, 1]; often skewed; consider robust estimation or a logit transform.
*spec_entropy*	Shannon entropy of the normalized 0–10 Hz spectrum.	Bounded; often non-normal.
*event_count*	Number of above-threshold transient-event intervals within the epoch.	Count variable; may be over-dispersed (consider robust SE or NB/quasi-Poisson).
*event_time_coverage*	Fraction of the epoch classified as “in-event.”	Bounded [0, 1]; often skewed.
*event_max_z*	Maximum robust z-score among detected transient events.	Extreme-value statistic; susceptible to artifacts/outliers.
*n_peaks*	Number of detected spectral peaks in 0–10 Hz (range 0–3).	Discrete; use count or non-parametric methods; report detection criteria.
*peaks_total_frac_power*	Total fraction of 0–10 Hz power explained by detected peaks.	Bounded [0, 1].
*total_power_dB*	Total integrated 0–10 Hz power (decibel scale).	Often near-normal after dB scaling; calibration-sensitive (state reference).
*bg_power_dB*	Background (non-peak) power level within 0–10 Hz (decibel scale).	Interpret jointly with *peaks_total_frac_power*; calibration-sensitive.
*bg_spec_entropy*	Shannon entropy of the background (non-peak) spectrum in 0–10 Hz.	Bounded; often non-normal.
*peak1_f0_Hz* ^a^	Center frequency of the dominant (largest) peak in 0–10 Hz.	Missing if no peak is detected; ensure consistent detection criteria.
*peak1_bw_Hz* ^a^	Bandwidth (e.g., − 3 dB width) of the dominant peak (Hz).	Positive and often right-skewed; specify the bandwidth method.
*peak1_frac_power* ^a^	Fraction of total 0–10 Hz power carried by the dominant peak.	Bounded [0, 1].
*peak2_f0_Hz* ^a^	Center frequency of the second-largest peak (if present).	Often missing/sparse; report *n* used.
*peak2_frac_power* ^a^	Fraction of total 0–10 Hz power carried by the second-largest peak.	Bounded [0, 1]; often sparse.
*peak3_f0_Hz* ^a^	Center frequency of the third-largest peak (if present).	Often missing/sparse; report *n* used.
*peak3_frac_power* ^a^	Fraction of total 0–10 Hz power carried by the third-largest peak.	Bounded [0, 1]; often sparse.
*RAW_mean*	Mean impedance over the raw 30 s epoch.	Continuous; sensitive to slow drift and calibration/electrode offsets; unsuited for uncalibrated cross-participant comparisons.
*LF_peak2peak*	Peak-to-peak amplitude of the low-frequency detrended signal $\:{x}_{lf}$ (*0–10 Hz*): $\:\mathrm{m}\mathrm{a}\mathrm{x}\left({x}_{lf}\right)\:-\:\mathrm{m}\mathrm{i}\mathrm{n}\left({x}_{lf}\right)$.	Outlier-sensitive; occasional excursions can inflate values; pair with robust measures (e.g., *IQR*).
*LF_iqr*	Interquartile range of $\:{x}_{lf}$ (75th–25th percentile).	Robust dispersion metric; typically, less outlier-sensitive than peak-to-peak.
*LF_meanAbsDeriv*	Mean absolute first difference of$\:\:{x}_{lf}$: $\:\frac{1}{N}\sum\:{x}_{lf}(n+1)\:-\:{x}_{lf}\left(n\right)$	$\:{x}_{lf}\:$is the low-frequency (0–10 Hz), linearly detrended impedance signal.
*LF_zeroCrossRate*	Zero-crossing rate of $\:{\mathrm{x}}_{\mathrm{l}\mathrm{f}}$ (proportion of sign changes; detrended about zero).	Bounded [0, 1]; can be sparse at very low oscillation rates; sensitive near zero (consider smoothing/hysteresis).
*LF_skewness*	Skewness (third standardized moment) of $\:{x}_{lf}$.	Can be unstable in small samples; outlier-sensitive; consider robust summaries/confidence intervals.
*LF_kurtosis*	Kurtosis (fourth standardized moment) of $\:{x}_{lf}$.	Outlier-sensitive; heavy tails may be artifact-driven; interpret with quality control.

For each feature, the table reports the signal‑processing definition and the statistical considerations

^a^Peak‑related features were computed only for the peaks that were detected in each trial. When one, two, or three peaks were present, the corresponding feature sets (*Peak 1*, *Peak 2*, *Peak 3*) were included; if a given peak did not exist, its associated features were coded as missing rather than estimated or imputed. Thus, *Peak 2* features were defined only when a second peak was detected, and *Peak 3* features only when a third peak was detected. This missingness reflects the true physiological absence of secondary or tertiary oscillatory peaks, not a failure of the detection procedure

### Statistical analysis

*Overview and analytical strategy.* We examined whether the 25 primary features set differed between the two Groups P and F, while accounting for repeated measurements across four static positions: *Tandem Standing Front*, *Stable Standing Position*, *Tandem Standing Behind*, and *Sitting Position*. The analysis combined model‑based repeated‑measures estimation using generalized estimating equations (GEE) [56], where activity was included as a within‑participant factor, ensuring that group effects were estimated after adjusting for systematic differences across the four positions. In addition, we implemented a participant‑level permutation test that required minimal distributional assumptions and preserved within-participant dependence through restricted exchangeability blocks [57]. For each permutation, the participant-Group labels (P/F) were randomly reassigned at the participant level (not at the individual trial level), thereby preserving the repeated‑measures structure across activities for each participant. This provided a non‑parametric validation of the GEE‑based findings. Statistical significance across the 25 primary features was evaluated using both Benjamini–Hochberg FDR [58] and Bonferroni corrections [59]. The dataset consisted of twenty-five participants (*P* = 14, F = 11), each assessed across the four activities, yielding 100 possible participant‑by‑activity observations (25 × 4); integrating the permutation test ensured robust inference against potential finite-sample biases inherent to standard GEE estimators in small-sample longitudinal designs [60].

*Missing data handling.* Analyses were performed feature‑wise using an available‑case approach: for each feature, only observations with non‑missing values for that feature were included in its corresponding model or test. This procedure provides a transparent, statistically appropriate way to accommodate feature‑specific data availability without altering the inferential framework. For higher‑order peak features, missingness was treated as structural (these quantities are undefined when the corresponding spectral peak is not present) and therefore reflects true peak‑structure variability rather than technical measurement failure.

*Primary model-based inference: GEE.* GEE provide a robust framework for analyzing correlated data from repeated measurements [56]. For each feature $\:{y}_{ij}$ (participant * i*, activity * j*), we fit a Gaussian GEE with identity link:

1 $$\:{y}_{ij}\:=\:{\beta\:}_{0}+{\beta\:}_{1}\cdot\:{\mathrm{CatP}}_{i}+\sum\:_{k}{\gamma\:}_{k}\cdot\:I({\mathrm{Activity}}_{ij}\:=\:k)+{\epsilon\:}_{ij}$$

Where $\:{\mathrm{C}\mathrm{a}\mathrm{t}\mathrm{P}}_{i}\:=\:1\:$for Group P and 0 for Group F, and $\:I(\cdot\:)$ are activity indicator variables (reference activity: TS_F) [61]. Participants were the clustering units with an exchangeable working correlation; inference used robust sandwich standard errors providing asymptotically consistent covariance estimates [56, 62] and a two-sided Wald test for $\:{\beta\:}_{1}\:$[61]. Because $\:{\beta\:}_{1}\:$represents the activity-adjusted mean difference between Groups, we report this effect as *Δ*(Group P − Group F) in all subsequent results. Furthermore, because some outcomes were bounded fractions or small counts, we treated the Gaussian identity link GEE as a robust marginal mean estimator and obtained p-values for $\:{\beta\:}_{1}$ from a participant-level permutation test that preserves the repeated measures structure (exchangeability blocks) [57, 63].

*Assumption-light validation: participant-level permutation testing.* Because several features exhibited non-normal distributions (e.g., skewness and heavy tails), we performed a participant-level permutation test for each feature to obtain an assumption-light p-value while preserving the repeated-measures structure. Permutation testing provides valid inference under minimal distributional assumptions [63, 64], making it particularly suitable for data with non-Gaussian characteristics. Permuting at the participant level, rather than at the observation level, preserves within-participant dependence across activities [57, 63] and yields a valid test under the null hypothesis of no difference between Groups P and F.

Permutation *p*-values were estimated using $\:{N}_{\mathrm{perm}}$ = 10,000 label permutations, and key inferences were confirmed using $\:{N}_{\mathrm{perm}}$ = 100,000 permutations to reduce Monte-Carlo error for borderline *p*-values [65]. Monte-Carlo permutation tests with 10,000-100,000 replicates provide sufficient precision for accurate *p*-value estimation, particularly for *p*-values near critical significance thresholds [57, 65]. For each feature:

1. Remove activity effects (residualization): compute the mean of the feature within each activity and subtract it from each participant-by-activity observation.
2. Aggregate within participant: compute the mean of residualized values within each participant to obtain one activity-adjusted value per participant.
3. Observed statistic: compute.
  2 $$\:{\varDelta\:}_{\mathrm{obs}}\:=\:{\bar{r}}_{P}-{\bar{r}}_{F},$$
  where $\:{\bar{r}}_{P}$ and $\:{\bar{r}}_{F}$ are the means of the participant-level residualized values in the Groups P and F, respectively.
4. Permutation: randomly permute participant‑Group labels (P/F) across participants (not across rows), keeping group sizes fixed within the set of participants contributing non-missing values for that feature, and recompute $\:\varDelta\:$ for each permutation.
5. Two-sided permutation *p*-value: with $\:{N}_{\mathrm{perm}}$ permutations, compute.
  3 $$\:{p}_{\mathrm{perm}}\:=\:\frac{\#\left(\mid\:{\varDelta\:}_{\mathrm{perm}}\mid\:\geq\mid\:{\varDelta\:}_{\mathrm{obs}}\mid\:\right)+1}{{N}_{\mathrm{perm}}+1}$$

By including the observed test statistic along with all permuted statistics when constructing the reference distribution, this approach ensures proper control of the Type I error rate and corresponds to the conventional method for deriving unbiased Monte Carlo *p*‑values [63, 66].

Furthermore, to ensure computational reproducibility, the Monte‑Carlo permutation procedure was executed using a fixed random seed [67]. This approach enforces deterministic pseudorandom number generation, ensuring that the sequence of permutations is identical across repeated analyses while preserving the statistical properties of the test [67].

*Multiple-comparison control (25 primary features).* To control false positives across the 25 primary features, we applied two complementary corrections separately to the GEE *p*-values and permutation *p*-values:

- Benjamini–Hochberg FDR: features were considered significant if $\:{q}_{\mathrm{FDR}}<0.05$.
- Bonferroni correction: features were considered significant if raw $\:p<0.05/25=\:0.002$ (equivalently, Bonferroni-adjusted * p<0.05*).

*Reporting and interpretation (pre-specified).* For each feature, we report the activity-adjusted difference between Groups P and F estimated using GEE ($\:{\beta\:}_{1}$; P − F), the corresponding $\:{p}_{\mathrm{GEE}}$ with Benjamini–Hochberg FDR and Bonferroni corrections, and the activity-adjusted permutation effect ($\:{\varDelta\:}_{\mathrm{obs}}$; P − F) with $\:{p}_{\mathrm{perm}}$ and corresponding corrections, together with Cohen’s *d* as a standardized measure of effect magnitude, computed from the participant-level activity-residualized values. In this study, permutation-confirmed results after multiple-comparison correction are treated as the most conservative evidence of group-associated differences, while GEE estimates are used to quantify effect direction and magnitude.

*Sensitivity to effect size.* Because the sample size was determined by recruitment, we report a sensitivity analysis rather than a post-hoc power calculation, which is uninformative when computed from observed effects. For independent groups of 14 and 11 participants at $\:{\upalpha\:}=0.05$ (two-sided) and 80% power, the smallest detectable standardized difference is $\:d\approx\:1.18$; a perfectly balanced split of the same total sample would yield $\:d\approx\:1.17$, indicating that the group imbalance has a negligible effect on sensitivity relative to the sample size itself. This two-sample approximation does not account for the repeated-measures structure or the activity adjustment used in the primary analysis, both of which increase precision, and is therefore conservative.

## Results

In this section, raw electrical EBI time series and representative time-frequency content were presented for Groups P and F, together with group-level power spectral density (PSD) summaries within 0–10 Hz. Subsequently, participant-adaptive spectral descriptors, the correlation structure among the 25 primary features, activity-adjusted Group differences estimated using repeated-measures GEE, and participant-level permutation tests were presented.

### Signal characteristics and spectral content

**Fig. 3:**
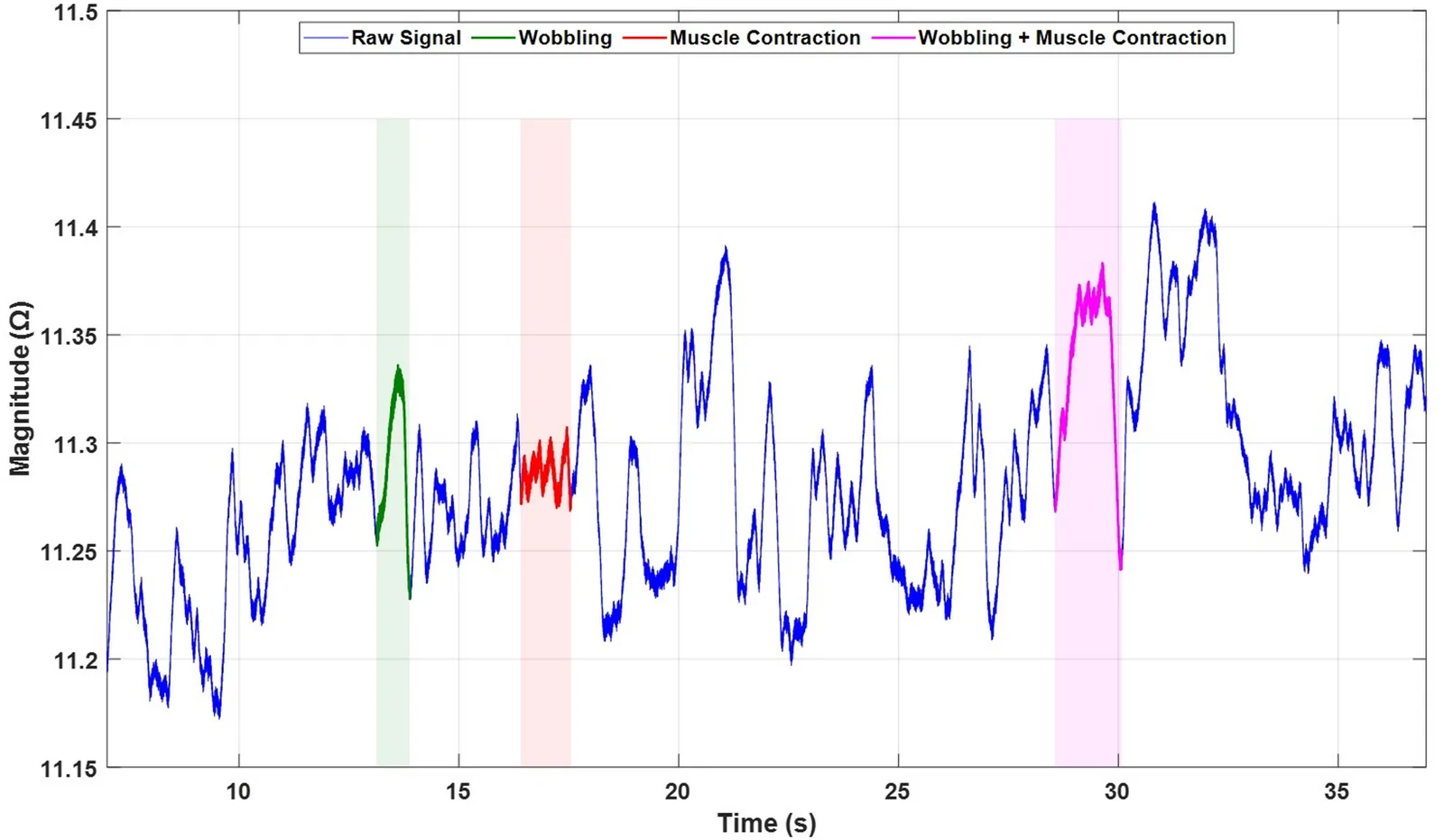
Raw EBI signal from a representative recording with annotated intervals: wobbling (green), EDL muscle contraction (red), and wobbling + contraction (magenta)

Researcher annotations were used to identify intervals of postural wobbling, EDL muscle contraction, and their co‑occurrence in the raw EBI trace. A representative recording with the annotated intervals is shown in Fig. 3. The trace fluctuates continuously around a slowly varying baseline, and the three annotated intervals illustrate the event types observed during the recordings. The wobbling interval (green) corresponds to a brief, single excursion of the signal. The contraction interval (red) shows a smaller-amplitude, more irregular segment without a comparable excursion. The interval in which both were observed (magenta) is the longest of the three and shows the largest deviation from the surrounding signal.

**Fig. 4:**
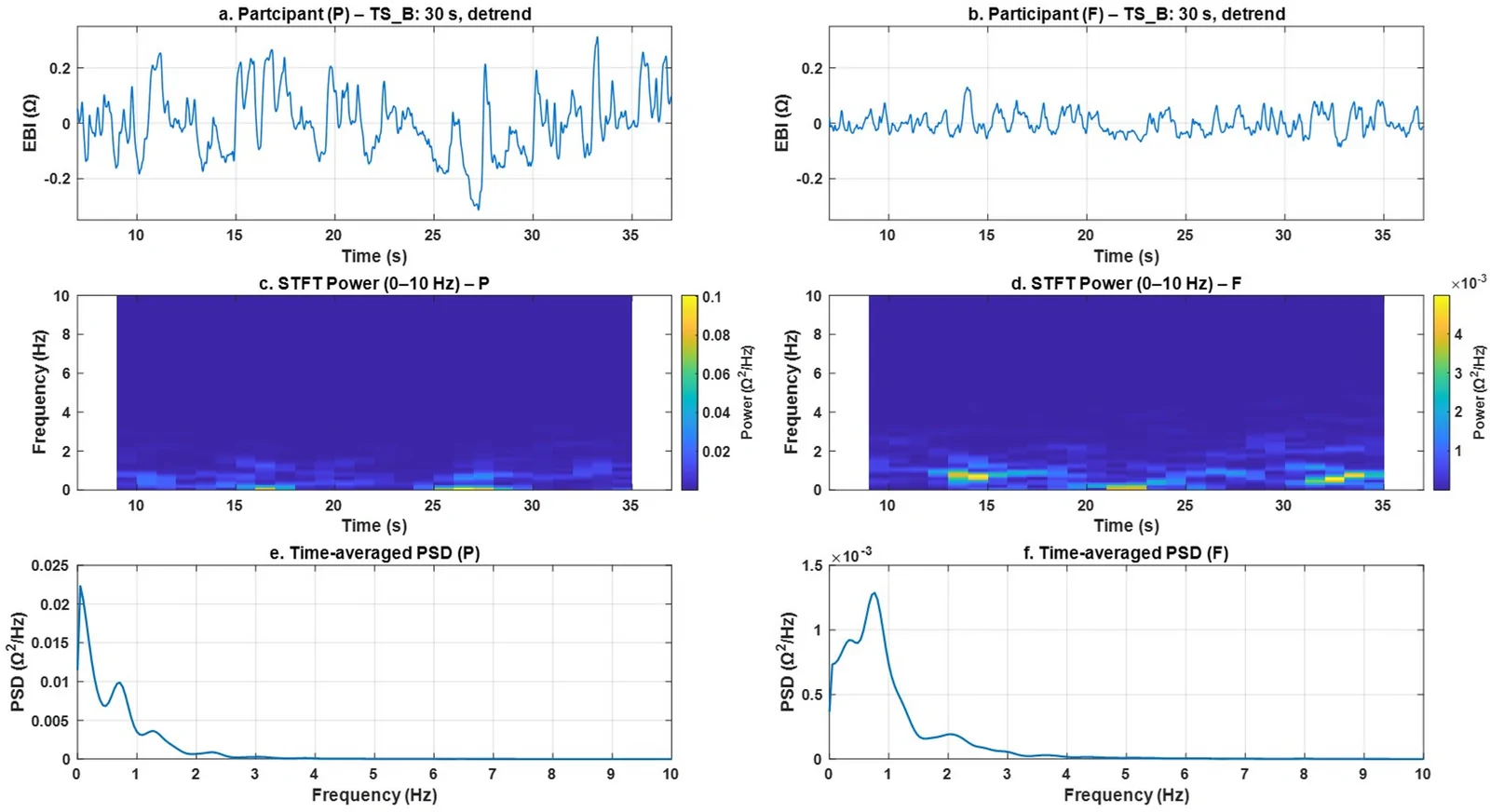
Representative 30 s EBI segments during TS_B. (**a**–**b**) Detrended time‑domain signals for one participant from Group P and one from Group F. (**c**–**d**) Short‑time Fourier transform power limited to 0–10 Hz. (**e**–**f**) Time‑averaged power spectral density for the same segments

A 30 s segment recorded during TS_B is displayed in Fig. 4. The detrended time‑domain signals for representative participants from Group P and Group F (panels a-b), the corresponding STFT power (0–10 Hz) (panels c-d), and the time‑averaged PSD (panels d-f) collectively show that the EBI signal energy during postural tasks is predominantly contained within 0–10 Hz, with negligible power above this band in the exemplars.

**Fig. 5:**
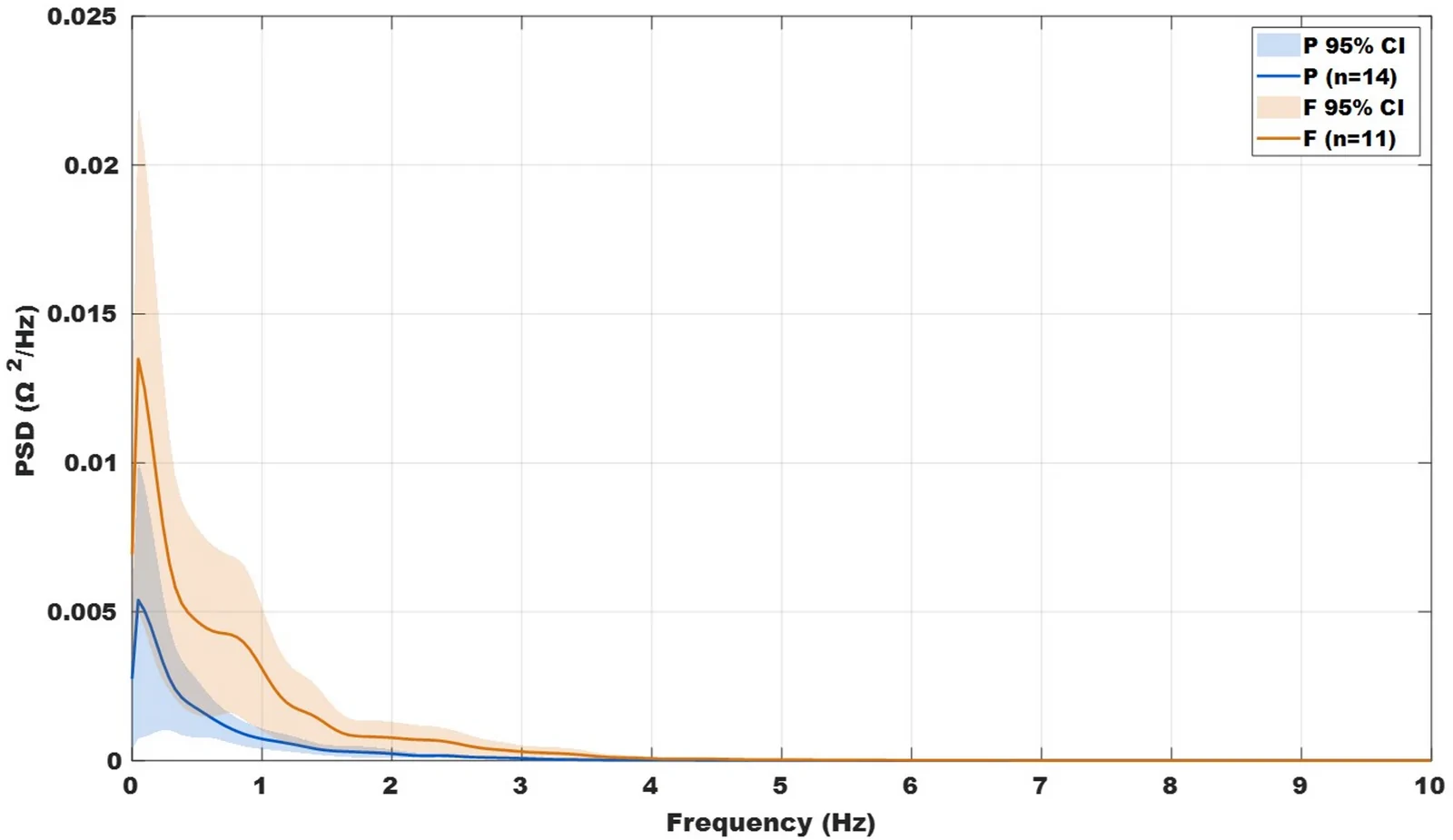
Group‑level PSD for 0–10 Hz with 95% confidence bands for Group P and Group F

Group‑level spectral energy distributions are summarized in Fig. 5 for Group P and Group F. Median PSDs with 95% confidence bands indicate that spectral power is concentrated below 10 Hz across participants in both cohorts. Subsequent analyses were therefore restricted to the 0–10 Hz band.

### Participant-adaptive spectral split

Within the 0–10 Hz band, we quantified (i) *frac_low*, the fraction of total 0–10 Hz power below the participant‑specific median frequency (*f_med_Hz*), and (ii) *f_med_Hz* (Hz). *frac_low* was part of the 25 primary features set used for primary inference, whereas *f_med_Hz* is reported descriptively to contextualize the participant‑adaptive split, and no multiplicity-controlled inference was performed for it.

**Fig. 6:**
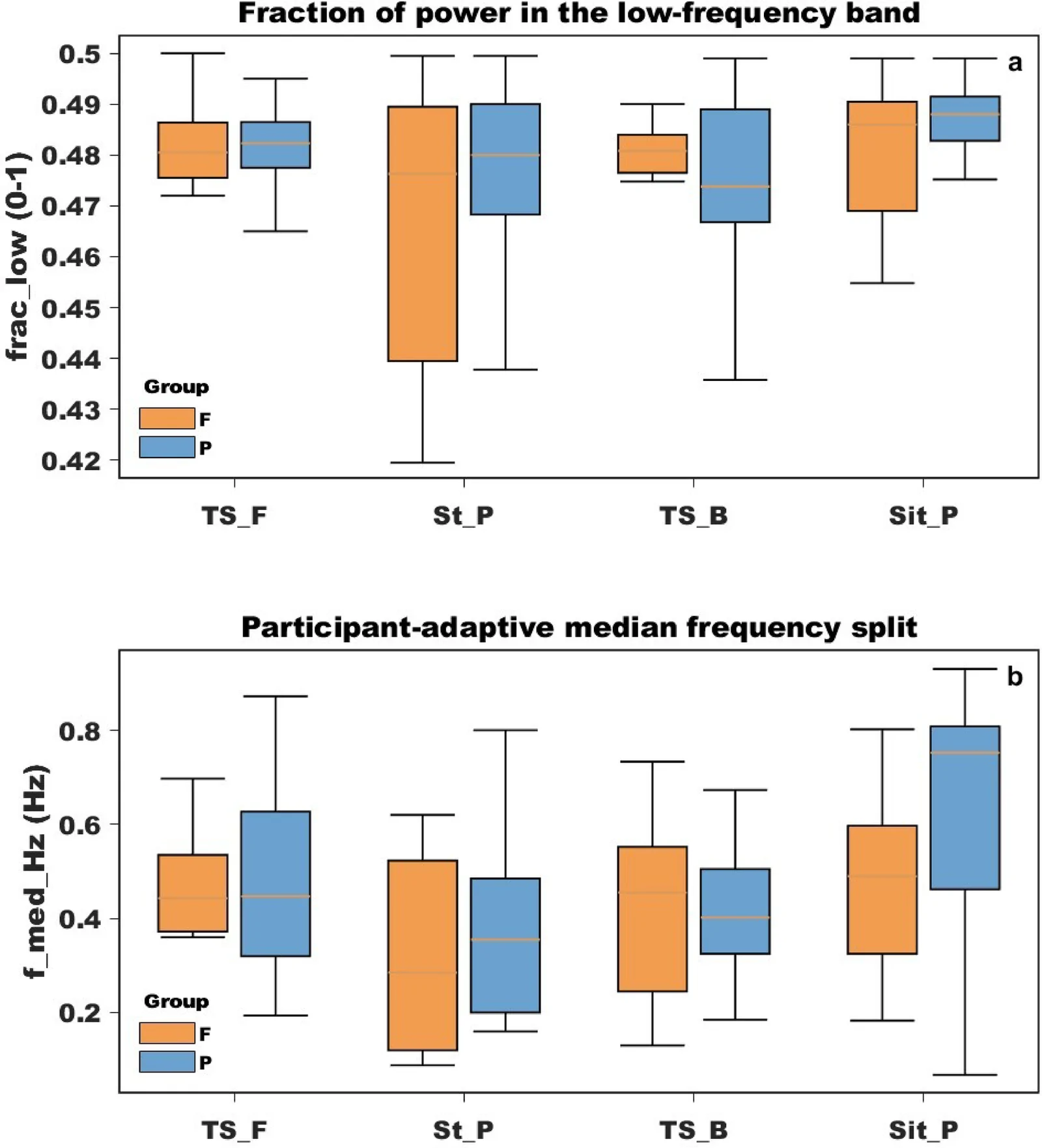
Participant‑adaptive spectral descriptors within 0–10 Hz by activity (TS_F, St_P, TS_B and Sit_P) and group (P and F). (**a**) *frac_low*: fraction of 0–10 Hz power below the participant‑specific median frequency. (**b**) *f_med_Hz* (Hz): participant‑specific median frequency that bisects integrated 0–10 Hz power. Boxplots show the median (line), interquartile range (box), and whiskers to 1.5×*IQR*

Across all four activities, *frac_low* was tightly clustered, with medians close to 0.48 and interquartile ranges overlapping between Group P and Group F (Fig. 6a). The participant-specific median frequency showed greater dispersion, both within and across activities (Fig. 6b). Median values of *f_med_Hz* remained below 1 Hz in every activity. The median split frequency was lowest during *St_P* and highest during *Sit_P*, whereas group-wise interquartile ranges overlapped within each activity.

### Correlation structure among 25 primary features

**Fig. 7:**
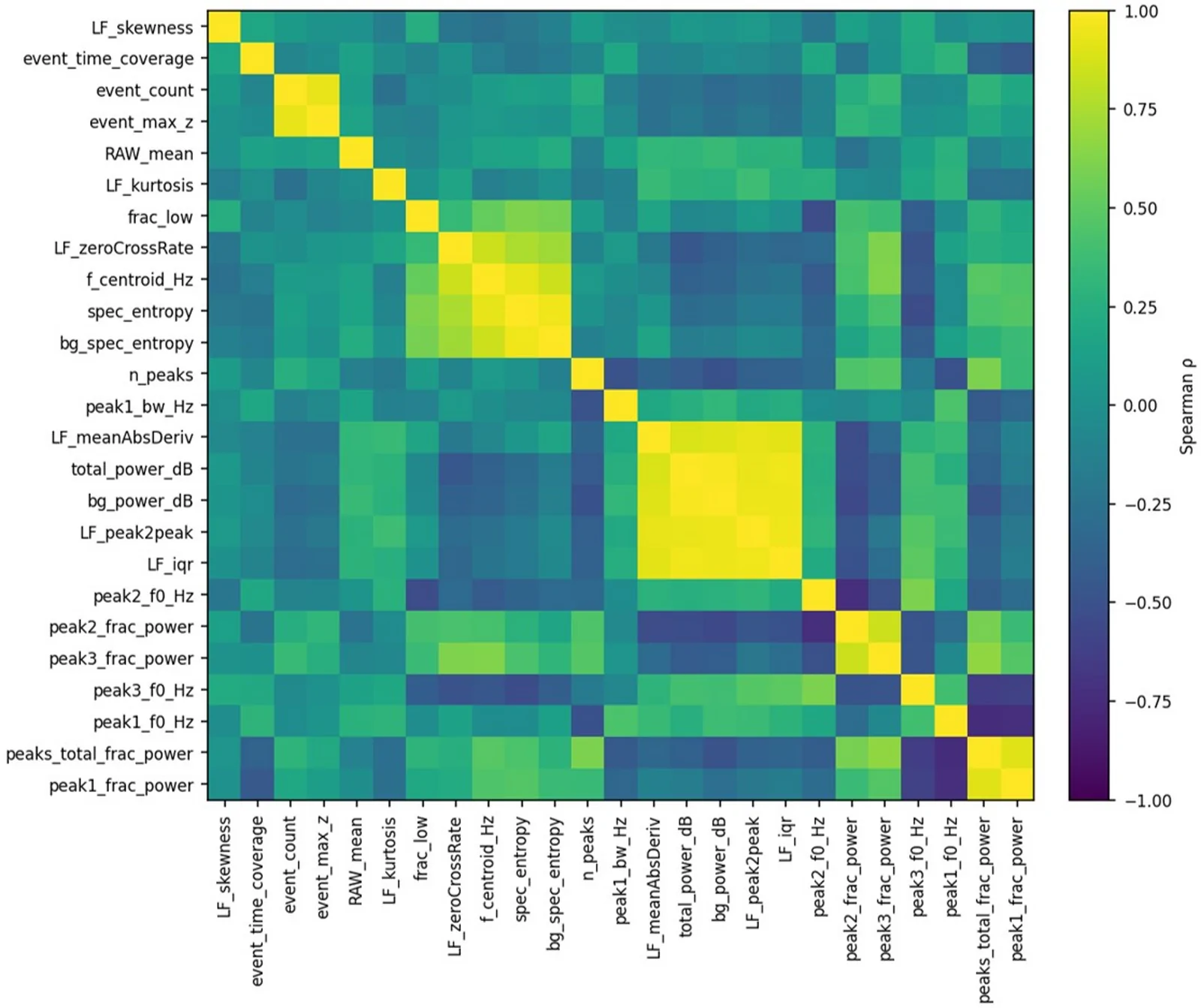
Spearman correlation (*ρ*) heatmap for the 25 primary features, computed at the participant level after activity residualization. Features are ordered by hierarchical clustering

Participant‑level, activity‑residualized Spearman correlations (*ρ*) were computed for the 25 primary features and are displayed in Fig. 7 (features ordered by hierarchical clustering). The heatmap showed two clusters: (i) an amplitude/roughness cluster including *total_power_dB*, *bg_power_dB*, *LF_iqr*, *LF_peak2peak*, *LF_meanAbsDeriv*; and (ii) a peak‑structure cluster including *peaks_total_frac_power*, *peak1_frac_power*, *peak1_f0_Hz*, *peak1_bw_Hz*, with higher‑order peak descriptors when present.

Using the pre‑specified flagging threshold $\:\left|\rho\:\right|\:>\:0.80$, 16 feature-pairs exceeded the cutoff. The strongest associations were: *total_power_dB* with *bg_power_dB* ($\:\rho\:\:=\:0.997$), *peaks_total_frac_power* with *peak1_frac_power* ($\:\rho\:\:=\:0.975$), and *LF_peak2peak* with *LF_iqr* ($\:\rho\:\:=\:0.974$). Additional strong correlations included *LF_iqr* with *LF_meanAbsDeriv* ($\:\rho\:\:=\:0.973$), *spec_entropy* with *bg_spec_entropy* ($\:\rho\:\:=\:0.940$), *LF_zeroCrossRate* with *f_centroid_Hz* ($\:\rho\:\:=\:\:0.923$), and *event_count* with *event_max_z* ($\:\rho\:\:=\:0.842$).

The number of observations differed across variables since analyses were performed feature‑wise. The proportion of missing observations ranged from 8% to 75% (median 8%, mean ≈ 19%). Higher‑order peak features (e.g., *peak3_f0_Hz*, *peak3_frac_power*) had greater missingness when secondary/tertiary peaks were undefined.

### Primary inference (P vs. F): activity‑adjusted repeated‑measures GEE

Primary analyses used Gaussian GEE with robust sandwich variance, adjusting for activity; effects are reported as the mean difference *Δ* (P − F) with 95% *CI*s. Among the 25 primary features, 10 showed nominal group differences ($\:{p}_{GEE}<\:0.05$). After multiplicity control across the feature family, three features remained significant ($\:{q}_{FDR}<\:0.05$); these same three also satisfied a Bonferroni familywise threshold ($\:raw\:p\:<\:0.002$). Per-feature observation counts varied (*n*_obs_ range: 25–92).

Significant estimates were: *peaks_total_frac_powe*r, *Δ* = 0.066 (95% *CI* 0.032–0.100; *p*_GEE_ = 0.000124; *q*_FDR_ = 0.0031; *n* = 92), *n_peaks*, *Δ* = 0.676 (95% *CI* 0.270–1.082; *p*_GEE_ = 0.00109; *q*_FDR_ = 0.0136; *n* = 92), and *peak1_frac_power*, *Δ* = 0.052 (95% *CI* 0.0197–0.0852; *p*_GEE_ = 0.00171; *q*_FDR_ = 0.0143; *n* = 75). All other features had $\:{q}_{FDR}\ge\:\:0.05$ and did not meet the Bonferroni threshold (Table 2).

**Table 2:** Complete list of features indicating survival across FDR and Bonferroni significance thresholds

Feature	*n*	Δ	95% CI	*p*_GEE_	q_FDR_
peaks_total_frac_power^*^	92	0.0664	(0.0325, 0.1004)	0.000124	0.0031
n_peaks^*^	92	0.676	(0.2703, 1.0817)	0.00109	0.0136
*peak1_frac_power* ^*^	*75*	*0.0524*	*(0.0197*,* 0.0852)*	*0.00171*	*0.0143*
*bg_power_dB*	92	−3.8828	(− 6.815, − 0.9505)	0.00945	0.0555
*peak1_f0_Hz*	75	−1.2479	(− 2.2111, − 0.2847)	0.0111	0.0555
*total_power_dB*	92	−3.5011	(− 6.3914, − 0.6108)	0.0176	0.0598
*LF_iqr*	92	−0.0417	(− 0.0763, − 0.0071)	0.0181	0.0598
*peak1_bw_Hz*	75	−0.2199	(− 0.4039, − 0.0359)	0.0191	0.0598
*LF_peak2peak*	92	−0.1181	(− 0.2234, − 0.0128)	0.0280	0.0736
*LF_meanAbsDeriv*	92	−0.000356	(− 0.000677, − 0.000036)	0.0294	0.0736
*f_centroid_Hz*	92	0.1359	(− 0.0232, 0.2949)	0.0940	0.206
*peak2_frac_power*	50	0.00683	(− 0.00128, 0.01494)	0.0989	0.206
*event_time_coverage*	92	−0.00191	(− 0.00438, 0.00057)	0.1310	0.2349
*spec_entropy*	92	4.5654	(− 1.3686, 10.4993)	0.1316	0.2349
*frac_low*	92	0.00512	(− 0.00552, 0.01575)	0.3457	0.5491
*bg_spec_entropy*	92	0.1292	(− 0.1435, 0.4019)	0.3532	0.5491
*LF_zeroCrossRate*	92	0.000508	(− 0.000611, 0.001627)	0.3734	0.5491
*peak3_frac_power*	25	0.00608	(− 0.0081, 0.02026)	0.4008	0.556
*peak2_f0_Hz*	50	−0.3992	(− 1.3749, 0.5765)	0.4226	0.556
*event_count*	92	0.1137	(− 0.1925, 0.4199)	0.4667	0.5834
*peak3_f0_Hz*	25	−0.424	(− 1.7765, 0.9284)	0.5389	0.6415
*event_max_z*	92	0.0978	(− 0.3651, 0.5607)	0.6787	0.7712
*LF_kurtosis*	92	0.0768	(− 0.4868, 0.6405)	0.7893	0.8382
*LF_skewness*	92	−0.0268	(− 0.2395, 0.1859)	0.8047	0.8382
*RAW_mean*	92	0.0914	(− 2.4247, 2.6075)	0.9432	0.9432

*Indicates features meeting both $\:{q}_{\mathrm{F}\mathrm{D}\mathrm{R}}<\:0.05$ and $\:{p}_{\mathrm{G}\mathrm{E}\mathrm{E}}<\:0.002$

### Assumption‑light validation: participant‑level permutations (activity-adjusted)

**Fig. 8:**
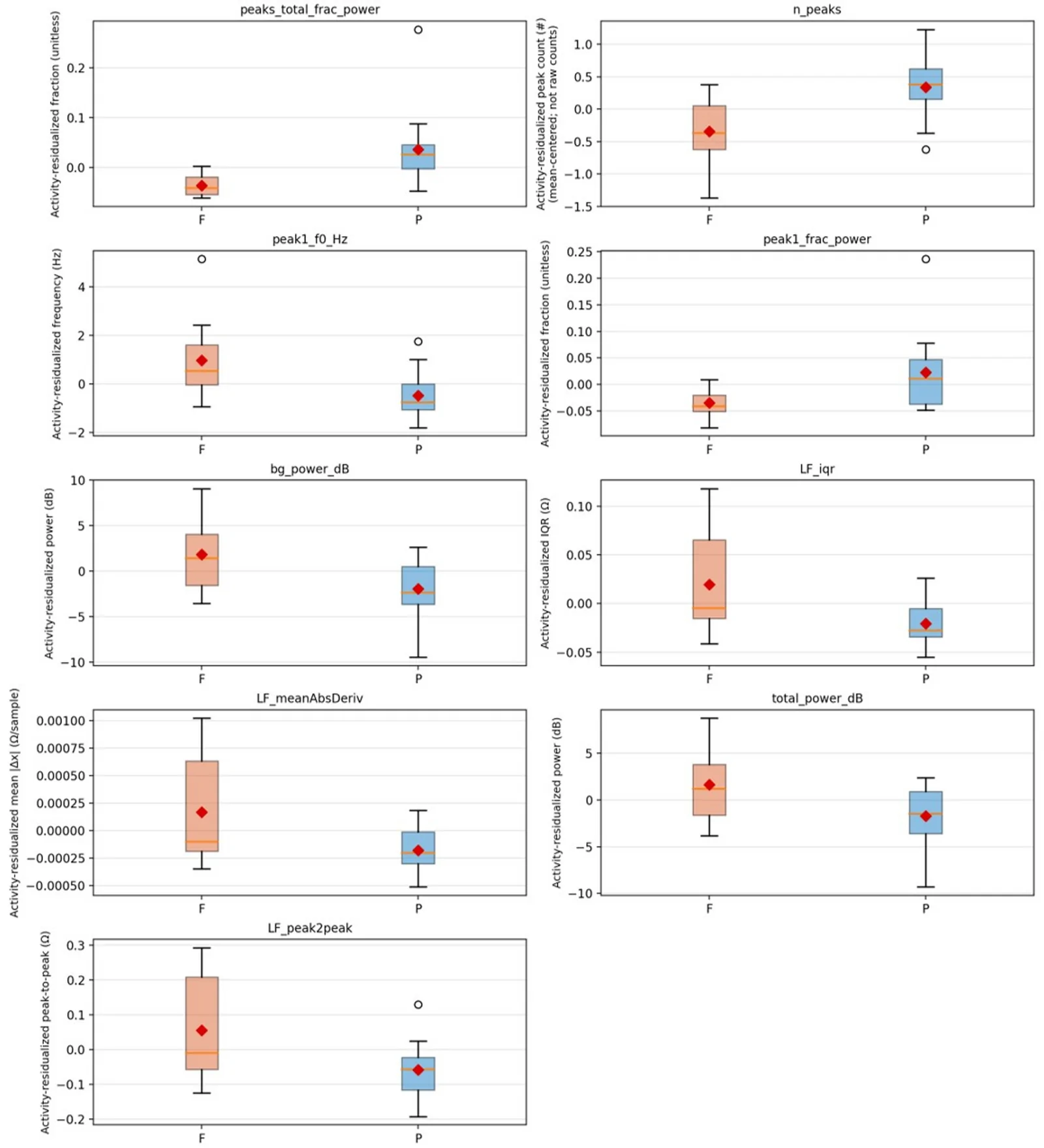
Participant-level, activity-residualized distributions (boxplots) for features with *p*_perm_ (100k) < 0.05. The box represents the interquartile range, the horizontal line indicates the median, whiskers extend to 1.5×*IQR*, and the red diamonds denote the group means

Participant‑level label permutations with activity residualization (100,000 permutations) were performed; effects are reported as the mean difference *Δ*_adj_ (P − F) and Cohen’s *d*. Among the 25 primary features, nine showed nominal evidence of group differences (*p*_perm_ (100k) < 0.05). After multiplicity control across the feature family (Benjamini–Hochberg FDR), two features remained significant, and one feature also met the Bonferroni threshold (raw *p* < 0.002). FDR‑significant features were: *peaks_total_frac_power* (*Δ*_adj_ = 0.0726; *d* = 1.15; *p*_perm_ (100k) = 0.00156; *q*_FDR_ (100k) = 0.039; *n* = 92) and *n_peaks* (*Δ*_adj_ = 0.683; *d* = 1.34; *p*_perm_ (100k) = 0.00314; *q*_FDR_ (100k) = 0.039; *n* = 92). Only *peaks_total_frac_power* satisfied the Bonferroni threshold. Results were unchanged with 10,000 permutations; we report 100,000‑iteration *p*‑values. Full results are provided in Table 3, and participant‑level residualized distributions for permutation‑nominal features are shown in Fig. 8.

**Table 3:** Participant‑level, activity‑residualized permutation tests for the 25 features

Feature	*n*	Δ_adj_	Cohen’s d	*p*_perm_ (100k)	q_FDR_ (100k)
peaks_total_frac_power^*^	92	0.0726	1.15	0.00156	0.039
*n_peaks*	92	0.683	1.34	0.00314	0.03925
*peak1_f0_Hz*	75	−1.452	−1.06	0.01003	0.06787
*peak1_frac_power*	75	0.0582	0.974	0.01251	0.06787
*bg_power_dB*	92	−3.7973	−1.06	0.01391	0.06787
*LF_iqr*	92	−0.0404	−1.02	0.01629	0.06787
*LF_meanAbsDeriv*	92	−0.0003	−0.949	0.02576	0.08394
*total_power_dB*	92	−3.3593	−0.944	0.02686	0.08394
*LF_peak2peak*	92	−0.1139	−0.93	0.03041	0.08447

Columns report *n* (observations), *Δ*_adj_, Cohen’s *d*, two‑sided permutation *p*‑value (100,000 permutations), and FDR-adjusted *q* (100,000 permutations)

*Indicates meeting both $\:{q}_{\mathrm{F}\mathrm{D}\mathrm{R}}\:\left(100\mathrm{k}\right)<\:0.05$ and raw $\:{p}_{\mathrm{p}\mathrm{e}\mathrm{r}\mathrm{m}}\:\left(100\mathrm{k}\right)<\:0.002$

## Discussion

Balance assessment is central to fall-risk evaluation in older adults, yet the tests most commonly used for this purpose require postures that themselves carry a risk of falling, and previous EBI studies, have relied on time-domain magnitude features during dynamic activities without examining balance ability during safe, static postures [30, 31]. Establishing whether muscle-level EBI carries balance-related information under such conditions is clinically relevant, because it would allow neuromuscular contributions to postural control to be assessed without provocative testing and would support repeated measurement in home-based and rehabilitation settings. In this study, we characterized the EBI signals during the static conditions sitting, standing, and tandem standing and showed that there are differences in the characteristics of the EBI signal depending on the individual’s balance ability assessed via single-leg stance on the same leg from which EBI was recorded. Building on these findings, this section addresses how the activity selection and experimental setup shaped the observable impedance dynamics, why time‑varying impedance fluctuations behavior required tailored signal‑processing methods, and why feature‑based descriptors were necessary to capture physiologically meaningful variability beyond total power alone. We also interpret key features in relation to underlying balance correction and, in a separate section devoted to physiological interpretation, consider how the observed differences between Groups P and F may relate to distinct balance-control strategies.

We selected static postures to isolate the portion of the EBI signal that exhibits low‑frequency impedance fluctuations likely reflecting subtle, balance‑driven muscle contractions, rather than the large, movement‑related changes that occur during tasks with substantial joint‑angle excursions, segment accelerations, and ground‑reaction force transients. Under static conditions, the observed low‑frequency impedance variations are less confounded by gross movement and can therefore be more plausibly related to the ongoing contractile activity used to maintain equilibrium [31, 68]. The EDL muscle was targeted because of its established role in ankle stabilization and small postural adjustments, particularly under narrow‑base tasks such as tandem standing [69]. Our previous work using the same setup showed that the EDL muscle exhibited distinct, reproducible impedance variations during static and dynamic activities, including characteristic fluctuations related to coordination and stability demands [30].

These prior findings support the suitability of EBI for capturing both large muscle activation and the subtle impedance changes associated with balance control. In practice, this required processing pipelines that preserve low-amplitude fluctuations while suppressing noise and baseline artifacts [70, 71], followed by feature extraction that quantifies amplitude, variability, and fluctuation content rather than relying on aggregate power alone [30]. The combined approach is intended to support a physiologically grounded interpretation of contraction-driven impedance dynamics at the muscle level, and the resulting descriptors are consistent with the muscle-specific signal patterns reported previously [30, 31].

Balance‑related impedance fluctuations are expected to vary over time, as postural control reflects a mixture of slow sway components and intermittent corrective adjustments rather than a single steady oscillation [72]. In such settings, reliance on a single global Fourier spectrum can obscure transient or posture-dependent spectral structure [72, 73]. Accordingly, we adopted a time-frequency representation (STFT) to summarize how spectral power evolves over the 30 s epoch, with window parameters held constant to ensure comparability across participants and activities. This choice is consistent with prior work in postural‑control signal analysis indicating that sway dynamics are not always well described by strict stationarity assumptions and can benefit from time-frequency characterization [73]. In parallel, bioimpedance recordings may exhibit slow baseline variability driven by factors such as electrode-skin interface dynamics, contact conditions, and gradual shifts in tissue–electrode coupling [71]. To avoid conflating absolute level with dynamic fluctuations, we separated the baseline level (raw mean impedance) from low‑frequency dynamics (0–10 Hz component after zero‑phase low‑pass filtering and linear detrending) [72, 74]. This preserved physiologically interpretable absolute level information while enabling extraction of drift‑robust descriptors of variability, roughness, and oscillatory structure. Finally, the deviation‑map event detection employed MAD‑based robust normalization to reduce sensitivity to heavy tails and occasional outliers (features common in physiological time series) [41], thereby improving the stability and comparability of transient descriptors across heterogeneous participants [75].

After establishing that the recorded time-series segments were of sufficient quality for analysis within the targeted 0–10 Hz band (a range that covers the low‑frequency content of posture‑related sway dynamics) [34, 76], we next characterized how spectral energy is distributed within this band before interpreting more specific feature families. This descriptive contextualization is important because apparent group differences in oscillatory metrics can, in some cases, reflect a simple global shift of power toward lower frequencies rather than a difference in spectral organization; isolating the background/aperiodic component from the periodic components is well established as essential for preventing their confounding [76]. We therefore summarized within-band power allocation using a participant-adaptive median-frequency split, reporting *frac_low* (the fraction of 0–10 Hz power below the participant-specific median frequency) together with *f_med_Hz* (the median split frequency; descriptive). The substantial overlap of *frac_low* between Group P and F across activities indicates broadly similar low–high power balance within 0–10 Hz, suggesting that any group-associated effects observed in subsequent analyses are more likely to reflect differences in spectral organization (e.g., peak structure and background composition) than a trivial redistribution of energy toward slower fluctuations. In parallel, the activity dependence of *f_med_Hz* provides context that posture modulates the characteristic tempo of impedance fluctuations, supporting the use of activity adjustment in the repeated-measures models [34, 72]. This step therefore reduces interpretive ambiguity and motivates the subsequent feature-level discussion.

After verifying signal and band‑level quality, we moved from the initial 61 features candidates to a set of 25 primary features (59% reduction) using pre‑defined rules that removed deterministic redundancy and limited the multiple‑comparison burden while preserving complementary information dimensions. Given the inherent coupling of biological systems, residual correlations among the primary features are expected and should be understood as physiological coupling rather than mathematical dependence. In this light, the post‑reduction correlation screen serves as interpretive context: when several features shift in the same direction, the pattern typically reflects a single physiological axis rather than multiple independent mechanisms. In our case, the retained set of primary features captures two complementary dimensions of EBI behavior during static balance: (i) magnitude/roughness of low‑frequency impedance fluctuations and (ii) spectral peak organization (i.e., how strongly fluctuations are concentrated into distinct peaks). Accordingly, clusters of effects within either dimension are expected and informative. Importantly, our inference strategy remained feature‑wise with multiplicity control across the primary feature set, so inter‑feature correlation does not bias the estimated group effects; instead, it supports transparent interpretation of partially overlapping constructs.

Across the 25 primary features, the most conservative evidence of category-associated differences is provided by features that both (i) survive multiple-comparison control and (ii) are supported by the participant-level permutation test that preserves the repeated-measures structure. Under this criterion, *peaks_total_frac_power* and *n_peaks* emerge as the most robust discriminators. In the activity-adjusted GEE analysis, two features (*peaks_total_frac_power* and *n_peaks*) showed clear separation, and both remained significant under participant-level permutation testing with multiplicity control (100,000 permutations). Both features were greater in Group P than in F, indicating that after adjusting for posture/activity, participants in Group P exhibited a larger fraction of 0–10 Hz power concentrated in discrete spectral peaks and a richer peak structure than participants in Group F. Collectively, these findings indicate that the low-frequency impedance dynamics in Group P are more strongly organized into discrete spectral peaks, whereas those in Group F are relatively more background- or aperiodic-dominated within the 0–10 Hz band. These statements describe the structure of the recorded signal; their possible physiological basis is considered separately below.

Features that reached significance in the activity-adjusted GEE framework but did not receive equally conservative permutation confirmation after multiplicity control (e.g., *peak1_frac_power*) remain informative for effect direction and magnitude, but should be interpreted as supportive rather than definitive, particularly given the modest sample size and non-Gaussian behavior of some descriptors. In this context, *peak1_frac_power* being greater in Group P is consistent with a stronger dominance of the primary oscillatory component in better-performing participants; however, this feature did not remain FDR-significant in the permutation analysis (100,000 permutations: *p*_perm_ (100k) = 0.0125, *q*_FDR_ = 0.0679), and is therefore best framed as hypothesis-supporting unless replicated or confirmed under stricter assumption-light criteria. Importantly, this difference in “GEE-confirmed vs. permutation-confirmed” does not imply that the GEE estimate is wrong; rather, it indicates that under a more conservative distribution-free validation with multiplicity control, the evidence for this specific feature is weaker than for *peaks_total_frac_power* and *n_peaks*.

A coherent secondary pattern, observed at nominal significance levels and consistent across multiple feature families, suggests that Group F tends to exhibit larger and more irregular low-frequency fluctuations. Specifically, *bg_power_dB* and *total_power_dB* tended to be greater in Group F in the activity-adjusted models (*bg_power_dB*: *Δ* = −3.88, *p* = 0.00945; *total_power_dB*: *Δ* = −3.50, *p*_GEE_ = 0.0176), and time-domain LF dynamics such as *LF_iqr*, *LF_peak2peak*, and *LF_meanAbsDeriv* also tended to be greater in Group F (*Δ* = −0.0417, *p*_GEE_ = 0.0181; *Δ* = −0.118, *p*_GEE_ = 0.0280; *Δ* = −0.000356, *p*_GEE_ = 0.0294, respectively). Because absolute power metrics depend on calibration and inter‑subject physiology (being influenced by subcutaneous fat/skin thickness, tissue water content, and electrode–skin interface impedance) we treat *bg_power_dB* and *total_power_dB* as supportive context rather than primary evidence [26, 70, 71]. Accordingly, our main inferences emphasize amplitude‑invariant “fractional” features (e.g., *peaks_total_frac_power*, *peak1_frac_power*), which are less sensitive to global scaling. These effects were also directionally consistent under permutation (all nominal *p*_perm_ < 0.05 at 100,000 permutations), but did not survive permutation-FDR correction, and should therefore be presented as convergent supportive context rather than as stand-alone primary claims. In combination, the pattern delineates a peak-dominated, smoother low-frequency signature in Group P and a more diffuse, larger-amplitude, rougher signature in Group F.

The interpretations offered in this section are exploratory. Because no concurrent EMG, inertial, or force-plate measurements were recorded, the physiological processes underlying the observed impedance differences cannot be identified directly, and the following account is presented as a plausible reading of the findings rather than as a demonstrated mechanism. Subject to this qualification, the results are consistent with established postural-control concepts: quiet stance is maintained through a combination of slow sway and intermittent corrective actions [77, 78], and differences in the regularity and organization of muscle contractions can manifest as differences in low-frequency spectral structure [34]. Within this framework, greater *peaks_total_frac_power* and *n_peaks* in Group P suggest a larger proportion of the impedance signal being organized into repeatable oscillatory components rather than diffuse broadband variability [76]. Conversely, a more background-dominated spectrum with greater fluctuation magnitude and roughness in Group F is compatible with larger, less regular corrective adjustments or greater variability in micro-movement and muscle activation patterns. As surface EBI reflects composite contributions from muscle geometry, fluid redistribution [26], and electrode–skin interface dynamics [71], modulated by activation and subtle motion, the observed differences cannot be attributed to a single physiological source on the basis of the present measurements.

Beyond their physiological interpretation, these feature relationships were internally consistent by construction. The features *peaks_total_frac_power*, *n_peaks*, and *peak1_frac_power* quantify how much of the 0–10 Hz spectrum is organized into discrete peaks, whereas *bg_power_dB* summarizes the complementary broadband component and therefore tends to vary inversely with peak-structure measures [76, 79]. Likewise, *total_power_dB* and LF dispersion/roughness measures (such as *LF_peak2peak*, *LF_iqr*, *LF_meanAbsDeriv*) quantify related aspects of fluctuation magnitude in frequency and time domains and were therefore expected to correlate because they reflect partially overlapping physiological constructs [71]. Because inferential testing was performed using feature-wise univariate models with multiplicity control across the pre-specified features, residual inter-feature correlation did not bias the estimated category effects, but it guides interpretation by indicating when multiple significant or suggestive features likely reflect a shared underlying physiological dimension rather than independent mechanisms.

Overall, the analysis is explicitly adjusted for posture/activity and preserved repeated measurements within participants (both in GEE and in participant-level permutation). Thus, the reported category-associated effects are not attributable to any single activity condition. Missing values in higher-order peak descriptors (e.g., *peak2*_* and *peak3*_*) largely reflect the structural absence of secondary/tertiary peaks in some trials [76, 80], and were handled feature-wise as specified.

Further consideration concerns the relationship between the grouping criterion and the measurement site. Participants were classified by single-leg-stance performance on the same leg from which EBI was recorded, so the group definition and the recorded signal share a limb, and the observed differences might in principle reflect properties specific to that limb rather than a general balance capacity. Two aspects of the design bear on this. The EBI recordings were not obtained during the single-leg-stance test but during four separate static postures, none of which reproduces the classification task, and the group effects were estimated with adjustment for activity, so they are not attributable to any single posture. The present design nonetheless cannot separate limb-specific muscle status from whole-person balance capacity, nor determine whether the observed features reflect a stable characteristic of the neuromuscular system or a state specific to the conditions studied.

This study has several limitations. (i) The sample size was modest, and Groups P and F were unequal and convenience-sampled, limiting power and generalizability and leaving potential residual confounding by age, sex, anthropometrics/body composition, and physical fitness. Participants were recruited exclusively from a fall-prevention training programme and were therefore already physically active and motivated to maintain their balance, which limits generalizability to less active or less engaged older adults. The unequal group sizes reduce efficiency only marginally relative to a balanced design of the same total size, but the modest sample means that effect estimates remain imprecise, as reflected in the confidence intervals reported in Table 2. Under limited power, effects reaching significance tend to overestimate the underlying difference, so the reported magnitudes should be regarded as provisional, with the direction and cross-posture consistency of the effects treated as the more reliable findings. (ii) The P/F participant‑Group label is a functional performance endpoint, not a direct mechanistic classifier. Although clinically meaningful, it may reflect multiple mechanisms (neuromuscular control, fatigue, strategy selection), and the present design cannot attribute observed impedance differences to a single mechanism. In addition, stance duration was recorded in 10 s intervals and the test was stopped at the 60 s criterion, so balance ability was available only as a binary contrast rather than as a graded measure; the strength of the association between EBI features and the degree of balance impairment therefore could not be quantified. (iii) Although signals were standardized to central 30 s windows and activity was modeled explicitly, results may be influenced by protocol and session factors (familiarization, fatigue, hydration, time of day); because each segment was recorded once and in a fixed order, any systematic effect of order or accumulated fatigue cannot be separated from the effect of posture, and test–retest reliability and between-session reproducibility were not assessed because they require a dedicated repeated-measures protocol with controlled electrode re-application. (iv) Surface EBI is sensitive to electrode placement and electrode-skin contact stability, and reflects mixed contributions from muscle, subcutaneous tissue, and the electrode interface, as well as subtle motion-related geometry changes; our baseline-dynamic separation and drift-robust processing mitigate but do not eliminate these influences, so tissue specificity cannot be assumed. (v) Only EBI was recorded; without concurrent reference measures (e.g., EMG and IMU/force-plate), mechanistic interpretation remains limited and multi-modal validation is warranted. (vi) Inference was performed feature-wise on the 25 primary features with multiplicity control; residual inter-feature correlation is expected due to shared physiology, and effect estimates may vary modestly with reasonable processing choices. Replication in a larger, demographically balanced cohort with standardized electrode placement, randomized segment order, and explicit reliability testing is needed to confirm robustness and support the generalization for the whole population.

These limitations primarily reflect study-design and measurement constraints rather than deficiencies of the analytical framework. Our processing and inference were selected to maximize robustness under these constraints; however, stronger mechanistic attribution and population-level generalizability will require multi-modal validation, reliability assessment, and independent replication.

Several directions would take these findings toward a community-based screening tool. The two permutation-confirmed features offer a compact starting point, though how many features a reliable index requires is an empirical question best settled in independent data; robustness would favour descriptors computable from every recording, since peak-structure features beyond the first spectral peak were not always defined. Recording stance duration continuously and to failure, rather than in fixed intervals with an upper cut-off, would allow balance ability to be treated as a graded measure and its relationship with EBI features examined as a dose-response rather than a group contrast. Recording EBI from both legs, and defining balance ability from a measure independent of either limb, would establish whether the discriminating features are limb-specific or reflect a general balance capacity, while repeated measurement across sessions would establish whether they are stable over time. Validation against instrumented posturography and established clinical balance measures, and ultimately against prospectively recorded fall outcomes, would establish clinical meaning, while a larger, demographically balanced cohort spanning a wider age range, including adults over 75 and followed prospectively, would test generalisation. Confirmed measurement stability under electrode re-application and an automated processing pipeline would then support unsupervised use.

Taken together, the activity‑adjusted analyses supported a compact P/F signature within the 0–10 Hz impedance dynamics. The most conservative findings pointed to stronger oscillatory organization in Group P, captured by greater *peaks_total_frac_power* and *n_peaks*, whereas Group F showed a relative shift toward less peak‑dominated, more diffuse background dynamics and, descriptively, larger and more irregular low‑frequency amplitude variations. This indicates that the functional separation is not simply a difference in overall power, but rather a difference in the organization of low‑frequency fluctuations [34, 76]. Group P exhibited more repeatable oscillatory structure, while Group F exhibited comparatively more irregular low‑frequency behavior. As discussed above, this pattern is compatible with less stable or less efficient balance control [81, 82], although that interpretation remains provisional in the absence of concurrent reference measures. Because effects were estimated with activity adjustment and participant‑level handling of repeated measures, this signature reflects group‑associated differences that are not attributable to any single activity condition.

In this study, we characterized surface EBI signals recorded during static balance tasks and identified a compact set of features within the 0–10 Hz band that may reflect balance-related phenomena such as subtle postural muscle contractions and low-frequency sway of the body. After adjusting for activity and accounting for repeated measurements, these features discriminated between Groups P and F, indicating that the groups differ in the organization of the impedance signal rather than in its overall magnitude. Because the protocol is performed in stable bilateral stance, it offers a practical alternative to more demanding assessments (e.g., single-leg stance), thereby reducing testing burden and potential fall risk in older adults while still providing information that may be clinically useful for assessing balance stability. Whether combining the most informative features yields a concise individual “signature” that can support screening or prediction of increased fall risk remains to be established; several directions toward this are outlined above.

In conclusion, this study’s results indicate that during static postures (sitting, stable standing, and tandem standing), frequency‑domain peak‑structure features of EBI distinguished balance ability in older adults. Low‑frequency spectral organization appears to provide sensitive markers, supporting the feasibility of EBI‑derived features as candidate muscle‑level biomarkers of balance‑control quality without requiring single‑leg standing during assessment. Future work should establish test-retest reliability, validate against instrumented balance references and prospective fall outcomes and evaluate whether multivariate combinations of these features yield compact individual-level signatures in larger, demographically balanced cohorts.

## Data Availability

Data availabilityThe data supporting the conclusions of this article will be made available by the corresponding author upon reasonable request.
